# Progress in Surgery

**Published:** 1896-07-25

**Authors:** 


### Progress in Surgery.

### ORTHOPÆDIC SURGERY.

**The Treatment of Postural Deformities of the Trunk** is the subject of a paper by Dr. Jacob Teschner.[1] The results in seventeen cases treated by him up to the present time show that fifteen were discharged cured and two very much improved. Of the fifteen cured cases all but one were rotary lateral curvatures of the spine of different degrees of severity. The average time of treatment was about two and a half months. He has devised a series of twenty-six exercises with dumb-bells, taking the standpoint that these deformities are due to (1) lack of strength and muscular development; (2) habitual faulty position with superimposed weight (3) loss of co-ordinating power; and (4) lack of muscular control. He corrects deformities by reversing these conditions, (1) by developing the strength of the muscles; (2) by acquiring an habitually corrected position with superimposed weight; and (3) by educating all the muscles to proper co-ordination and complete control.

**Lateral Curvature of the Spine.**—Dr. R. H. Sayre[2] discusses infantile paralysis as a cause, and says that slight differences in the deep muscles about the spine are capable of producing lateral curvature, and yet they are difficult to detect. He adds that German writers say that in lateral curvature due to paralysis the bulging is always on the paralysed side. His experience, however, does not bear out this statement. He then goes into the matter of an efficient scoliosometer, and describes an arrangement of Dr. Jacobi, by means of which the amount of deformity may be estimated by its effects on the shape of the thorax. The arrangement is a very simple one. Two lead tapes hinged together are moulded carefully to the entire circumference of the thorax, the hinge being on the spine. The lead is then removed by opening the hinge, and, after closing it again, it is placed on a level board, and the space enclosed by it is filled with moist plaster of Paris. By this means a thin plaster cast representing the horizontal section of the thorax is obtained.

**Congenital Absence of Both Radii.**—A. E. Taylor.[3] The subject was three years of age. The forearms were quite short and markedly bowed, with the concavity outward. The muscles on the radial aspects were poorly developed. From the wrist joints the hands were turned outward at an angle of about 50 degrees. The wrist-joints themselves were loose and freely movable, and the ligaments appeared to be lax. Pronation and supination could not be performed, but the hands could be imperfectly rotated from the shoulders. Flexion and extension were normal. In addition, on the left hand there was no thumb, while the right thumb consisted only of two phalanges, the metacarpal bone being suppressed.

**Congenital Displacement of the Hip-Joint.**—Much attention has been devoted to this subject in various periodicals during the past three months, and a perusal of the literature leads to the conviction that the success of operative measures is by no means assured, and the methods of recumbency and extension with abduction are to be preferred whenever it is possible. Mikulicz[4] has used with success an apparatus by which extension is applied to the limb in an abducted and everted position. The apparatus is only applied during ten or twelve hours a-day, but at other times pressure is kept up on the trochanters by a special corset; the full degrees of extension, abduction, and external rotation are gradually reached in about three months. Of five children varying in age from four months to four and a-half years, in whom this treatment was adopted, three were cured and two much improved. In the three completely successful cases, the head of the femur was so firmly fixed in the acetabulum, that it could not be displaced by a full flexion, adduction, and internal rotation. Schede[5] finds that in children who have not yet walked, reduction can be effected by simple traction and slight abduction, and that the head of the femur can be retained in position by moderate pressure on the trochanter. Even after the second or third year, when secondary changes in the bones have occurred, reduction can be effected by continuous extension for a few weeks or months, combined with abduction and pressure over the trochanter. Lorenz[6] has devised a form of mechanical treatment which has proved most successful in suitable cases. The actual reduction is effected under anæsthesia by the careful use of a screw-extension apparatus, which is applied to the limb in a strongly-abducted position, whilst counter extension is made by a perineal band. When the top of the trochanter has been drawn down to its normal level or even below it, extension is kept up for a few minutes in order thoroughly to stretch the soft parts. Whilst extension is still maintained an attempt is now made by extreme abduction to engage the head of the femur beneath the upper border of the acetabulum. If necessary, the adductors may be divided at this stage. Hoffa,[7] in his later cases, has endeavoured to spare the muscles as much as possible, and gives the results obtained in 112 operations performed on 82 patients, in 30 of whom the dislocation was bilateral. In all, there were seven deaths; three from inter-current diseases (pneumonia, intestinal catarrh, and diphtheria), three as the direct effect of prolonged operation in young children, and one possibly from iodoform poisoning. The last 47 operations were performed without a single accident. In nine cases ankylosis occurred, and in eleven there was recurrent dislocation. In the remaining cases the results were satisfactory, and the improvement steadily increased; the affected limb was lengthened and in the bilateral cases the lordosis and waddling gait were much lessened. Gibney,[8] in a clinical lecture, discusses the subject, and believes the displacement to be due to the non-development of the upper part of the rim of the acetabulum. One case is described by him which has been treated by continuous traction for over two years, and he says, "I am pretty well convinced that the head will stay where I put it, but I dare not take off the splint yet. I began at eighteen months of age, and now I use a walking splint with a joint at the knee." Speaking of Hoffa's operation, Dr. Gibney adds: "I have seen a number of his results. Though they were all recent, they were most commendable. The age most suited for his operation is between two and five years; cases beyond nine or ten do not do well. There is too much violence required to get the head of the bone into position." With reference to Hoffa's operation, the inventor[9] of it says, "the object must be something less than a complete *restitutio ad integrum,* and this is true both as regards the position of the limb and its mobility. It is usually convenient to make the new cotyloid cavity somewhat in front of the normal position and also higher. It is not to be expected that the full result can be obtained immediately after operation, however good the operative result. The functional result must depend largely on the results of after-treatment by exercises and massage." Dr. T. Halsted Myers[10] has collected 302 cases of Hoffa's operation, and found that the mortality has been between 3 and 4 per cent. The latest reports were much better. There has been no death in the last 22 cases of Broca or in the last 100 cases of Lorenz. The danger of the operation seems to be dependent almost entirely on sepsis, therefore the utmost care in this respect was necessary. The manipulations of Paci should first be tried as if to reduce a traumatic dislocation, and the limb fixed in an abducted position. After three or four months the patient may be allowed to walk with a protective splint. If this treatment fails after a fair trial, Lorenz's operation was indicated. Dr. Myers then showed a girl who had been cured by him. The dislocation was reduced by Paci's method, and the joint subjected to rather severe manipulations with the hope of exciting adhesive inflammation. The limb was fixed by a plaster of Paris in an abducted position without extension, as he did not believe that extension was indicated if the muscular retraction had been fully corrected at the operation. The spica had been removed about once a month, and the joint manipulated and again fixed in an abducted position. But it seems the case is too early to report as a cure. Kirmisson,[11] speaking of the duration of operation, remarks, Lorenz alleges that it only requires ten minutes to perform in easy cases and twenty minutes in more complicated cases. On careful perusal of his observations, however, Kirmisson has found thirty-seven cases in which the operation lasted half-an-hour or more, and nineteen others in which it lasted three-quarters of an hour. As a matter of fact, Kirmisson thinks that the operation takes on an average twenty-five or thirty minutes in simple, and three-quarters of an hour in difficult cases. It is consequently a serious operation, especially in very young children. In spite, therefore, of the great improvement recently made in the operation for the relief of congenital displacement of the hip, it cannot be considered as entirely benign, while the results are frequently indifferent and sometimes decidedly bad. For this reason he is inclined to side with those who accord to orthopædic measures a large share of the treatment of this affection. If children are treated at a very early date, not by operation, but by orthopædic measures, excellent results may be confidently looked for.

**Coxa Vara.**—Professor Ogston,[12] after briefly discussing the literature of the affection, states that the following are its characteristics, namely: gradual onset of the affection in one or both hip-joints accompanied with knee-pain, limping and wasting of the limb, shortening, eversion, and partial or entire loss of the power of abduction. In the diagnosis, emphasis is laid on the co-existence of eversion and adduction, these symptoms never being simultaneously present in hip-joint disease, for which coxa vara is often mistaken. Professor Ogston gives drawings of three cases, and insists upon the value of Bryant's ilio-femoral triangle in estimating the degree of the affection. Kraske,[13] of Freiburg, has attempted to remedy the deformity by removing a wedge of bone from the neck of the femur. The base of the wedge is directed forwards and upwards, and it is chiselled out of the bone at the junction of neck and trochanter.

**Genu Recurvatum.**—Camperon[14] notes the frequency of this affection in coxitis in young subjects, and believes it to be due to the position in which the limb is placed and kept while the child is lying in bed with the extension on. He advises that the limb, instead of being forcibly extended, should be lightly flexed.

**Ricketty Deformities of the Long Bones and their Treatment.**—R. W. Parker[15] goes into the question of osteotomy, and thinks that not all cases of persistent ricketty deformity are suitable for operation. The lower limbs in their totality may be considerably deformed, such deformity being made up of curves variously distributed. For such cases, not one, but several osteotomies would be necessary. The cases best suited for operation are those where the deformity is confined to one bone and is of a simple kind. Osteotomy is thus limited in its application, and the more Parker sees of rickets, the less he inclines to its adoption.

**Flat-foot.**—R. W. Lovett and John Dane[16] first challenge the general opinion that the infantile foot is flat, and say that the apparent flatness is due to a pad of fat placed beneath the arch. They carefully distinguish between the pronated foot or the painfully spasmodic foot, and simple flat-foot of feeble muscular development. A. H. Tubby[17] discusses the treatment of flat-foot. General treatment is first spoken of, including the relief of pain. For local treatment the means we have at our disposal are: Rest, active and passive exercises, mechanical support and operative measures, including forcible manipulation. But the exact line of treatment must depend upon the degree of severity and duration of the deformity. Briefly, it may be said that in moderate degrees of flat-foot rest, exercises, and mechanical support should all be employed, but each to an amount varying with the special causes at work and the condition of the muscles and ligaments. For the severer degrees tenotomy and forcible wrenching are advocated.

**Arthrodesis.**—Dr. Isnardi[18] contributes two cases of arthrodesis of the ankle for paralytic club-foot. The results were excellent.

**Club-foot.**—A. H. Tubby[19] gives a method of examining club-feet. The various steps are: (1) The history; (2) the gait on entering the room; (3) the position of the foot and limb on standing and sitting; (4) an outline or impression of the sole of the foot; (5) general examination of the affected limb or limbs as to shape, size, muscular development, diminished or excessive mobility of the joints, temperature, the condition of the skin as to colour, integrity, and the presence of corns or thickened skin over the heels and beneath the balls of the toes (the boots should also be looked at, and any unequal wear at one or more spots noted); (6) the passive movements which may be effected by the surgeon and the directions from which resistance is felt; (7) localisation of the resistant structures; (8) localisation of contracted or paralysed muscles (this is effected by touch and by movement on the part of the patient); (9) electrical reactions; and (10) signs of abnormal and arrested development, especially of bones. The treatment of club-foot is discussed by Lewis Sayre[20] under the title of the early management of congenital club-foot, who advocates immediate operation after birth; also by R. H. Sayre[21] who, in severe cases, prefers gradual measures to astragalectomy.

**Hallux Valgus.**—Dr. Delbet[22] advises resection of the head of the metatarsal bone together with pulling the extensor longus pollicis tendon inwards, and fixing it in its new position by means of a flap of periosteum.

[1] N. Y. Med. Record. [2] Internat. Clinics, IV.. 4. [3] Internat. Med. Mag., March, 1896. [4] Archiv. für Klin. Chirurgie. Bd. xlix. Hft. 2. [5] Verhandlungen der deutschen Gesellschaft für Chirurgie. XXIII. Kongress; and Annals of Surgery, 1895, vol. 1, p. 348. [6] Centralblatt für Chirurgie, 1895, pp. 153 and 761. [7] Archiv. für Klin. Chirurgie, Bd. li., 1895. [8] Internat. Clinics IV. 3. [9] Berliner Klinik, June, 1895. [10] N. Y. Med. Jour., March 28, 1896, and Amer. Medico-Surgical Bull., April 11, 1896. [11] Medical Week, April 24, 1896. [12] The Practitioner, April, 1896. [13] Centralblatt für Chirurgie, Feb. 8, 1896. [14] N. Y. Med. Record, March, 1896. [15] Internat. Clinics, IV., 3. [16] N. Y. Med. Jour., March, 1896. [17] The Hospital, March 28 and April 4, 1896. [18] Centralblatt für Chirurgie, March 21, 1896. [19] The Lancet, January 18, 1896. [20] N. Y. Med. Record, April, 1896. [21] Amer. Medico-Surg. Bull., December 15, 1895. [22] Medical Week, Feb. 26, 1896.

